# Profiling and bioinformatics analyses reveal differential circular RNA expression in radioresistant esophageal cancer cells

**DOI:** 10.1186/s12967-016-0977-7

**Published:** 2016-07-28

**Authors:** Huafang Su, Fuqiang Lin, Xia Deng, Lanxiao Shen, Ya Fang, Zhenghua Fei, Lihao Zhao, Xuebang Zhang, Huanle Pan, Deyao Xie, Xiance Jin, Congying Xie

**Affiliations:** 1 Department of Radiotherapy and Chemotherapy, The First Affiliated Hospital of Wenzhou Medical University, No. 2 Fuxue Lane, Wenzhou, 325000 China; 2 Department of Thoracic Surgery, The First Affiliated Hospital of Wenzhou Medical University, Wenzhou, 325000 China

**Keywords:** Radioresistance, Esophageal cancer, Circular RNA, Microarray

## Abstract

**Background:**

Acquired radioresistance during radiotherapy is considered as the most important reason for local tumor recurrence or treatment failure. Circular RNAs (circRNAs) have recently been identified as microRNA sponges and involve in various biological processes. The purpose of this study is to investigate the role of circRNAs in the radioresistance of esophageal cancer.

**Methods:**

Total RNA was isolated from human parental cell line KYSE-150 and self-established radioresistant esophageal cancer cell line KYSE-150R, and hybridized to Arraystar Human circRNA Array. Quantitative real-time PCR was used to confirm the circRNA expression profiles obtained from the microarray data. Bioinformatic tools including gene ontology (GO) analysis, KEGG pathway analysis and network analysis were done for further assessment.

**Results:**

Among the detected candidate 3752 circRNA genes, significant upregulation of 57 circRNAs and downregulation of 17 circRNAs in human radioresistant esophageal cancer cell line KYSE-150R were observed compared with the parental cell line KYSE-150 (fold change ≥2.0 and *P* < 0.05). There were 9 out of these candidate circRNAs were validated by real-time PCR. GO analysis revealed that numerous target genes, including most microRNAs were involved in the biological processes. There were more than 400 target genes enrichment on Wnt signaling pathway. CircRNA_001059 and circRNA_000167 were the two largest nodes in circRNA/microRNA co-expression network.

**Conclusions:**

Our study revealed a comprehensive expression and functional profile of differentially expressed circRNAs in radioresistant esophageal cancer cells, indicating possible involvement of these dysregulated circRNAs in the development of radiation resistance.

**Electronic supplementary material:**

The online version of this article (doi:10.1186/s12967-016-0977-7) contains supplementary material, which is available to authorized users.

## Background

Esophageal cancer is the eighth most frequently diagnosed and the sixth highest mortality rate cancer in the world [1]. The 5-year survival rate of esophageal cancer patients with localized disease is less than 20 % [2]. Radiation therapy (RT) plays a crucial role in the treatment of esophageal cancer [3]. Although complex multidisciplinary methods incorporating surgery, chemotherapy, and radiotherapy had been applied in the treatment of esophageal cancer, the rate of local recurrence and distant metastasis remains high [4, 5]. Radiotherapy resistance has been considered as one of the most important reasons for local tumor recurrence or treatment failure [6].

Circular RNAs (circRNAs), unlike the well known linear RNA, forms a covalently closed continuous loop [7, 8]. CircRNAs are integral, conserved, and demonstrated to be resistant to RNase R treatment [9–11]. With the advent of novel biochemical and computational approaches, circRNAs have been represented as a research hotspot in the RNA field [12]. Accumulating evidence demonstrated that circRNAs involve in the development of several types of diseases, such as Alzheimer’s disease [13], atherosclerotic vascular disease [14] and cancer [15, 16]. Recent studies found that circRNAs can function as microRNAs (miRNAs) sponges [17, 18], RNA-binding protein sequestering agents, and nuclear transcriptional regulators. The recently identified circRNA, ciRS-7, which acts as a designated miR-7 sponge, involves in competing in endogenous RNA networks [19].

We previously developed a radioresistant esophageal squamous cancer cell line (KYSE-150R) by irradiating esophageal cancer cells KYSE-150 with gradient dose [20–22]. To explore the underlying molecular regulation mechansim of circRNAs in the radioresistance, circular RNA microarray was used to detect the differential expressed circRNAs between radioresistant esophageal cell line KYSE-150R and the parental cell line KYSE-150. Our results suggested that the aberrant expression of circRNAs may play a role in transformation of radiation resistance of esophageal cancer cells.

## Methods

### Cell culture and reagents

Human esophageal squamous cancer cell lines KYSE-150 were purchased from the American Type Culture Collection (Manassas, VA, USA). Radioresistant cell line KYSE-150R has been previously established in our department by gradient dose irradiation treatment. Both KYSE-150 and KYSE-150R were cultured in RPMI-1640 (Gibco, Life Technologies Inc., Grand Island, NY, USA) with 100 unit/ml of penicillin, 100 mg/ml of streptomycin, and 10 % fetal bovine serum at 37 °C in a humidified incubator containing 5 % CO_2_. The cell lines were sub-cultured every 2–3 days following digestion at room temperature with 0.5 ml trypsin/EDTA per well (Sigma-Aldrich Ltd, UK).The viability was reported as the percentage of the viable cells number to the total cells number. There was an average viability over 95 %, determined by Trypan Blue staining.

### RNA isolation, purification and hybridization

Three samples were collected from each of the two cell cultural groups and used for the following RNA extraction. Total RNA from each sample was treated with Rnase R (Epicentre, Inc.) to remove linear RNAs and to enrich circRNAs. Then, the enriched circRNAs were amplified and transcribed into fluorescent cRNA utilizing a random priming method (Arraystar Super RNA Labeling Kit; Arraystar). The labeled cRNAs were purified by RNeasy Mini Kit (Qiagen). The concentration and specific activity of the labeled cRNAs (pmol Cy3/μg cRNA) were measured by NanoDrop ND-1000. One μL of each labeled cRNA was fragmented by adding 5μL 10× Blocking Agent and 1 μL of 25× Fragmentation Buffer, and then the mixture was heated at 60 °C for 30 min. Finally 25 μL 2× Hybridization buffer was added to dilute the labeled cRNA. Hybridization solution of 50 μL was dispensed into the gasket slide and assembled to the circRNA expression microarray slide. The slides were incubated for 17 h at 65 °C in an Agilent Hybridization Oven. The hybridized arrays were washed, fixed and scanned using the Agilent Scanner G2505C.

### Microarray data analysis

Scanned images were imported into Agilent Feature Extraction software (version 11.0.1.1) for raw data extraction. Quantile normalization of raw data and subsequent data processing were performed using the R software package. After quantile normalization of the raw data, low intensity filtering was performed. The circRNAs with at least 3 out of 6 samples flagged in “P” or “M” (“all targets value”) were retained for further analysis. When comparing the profile differences between two groups (such as disease versus control), the “fold change” (i.e. the ratio of the group averages) between the groups for each circRNA was computed. The statistical significance of the difference may be conveniently estimated by t test. CircRNAs having fold changes ≥2 and *P* values <0.05 were selected as of significantly differential expression.

### Real-time PCR validation

Quantitative Real-time PCR was used to confirm the circRNA expression profiles obtained from the microarray data. Total RNA was extracted from cells using Trizol Reagent (Invitrogen) and reversely transcribed into cDNA using Super Script TM III Reverse Transcriptase (Invitrogen) according to a standard protocol. The relative gene expression was determined using ViiA 7 Real-time PCR System (Applied Biosystems). All samples were normalized to the signal generated from GAPDH (Sangon Biotech, Shanghai, China). Data was shown as fold change (2−ΔΔCt) and analyzed initially using Opticon Monitor Analysis Software V2.02 (MJ Research, Waltham, MA, USA). Triplicates were performed for each sample in three independent experiments. Primer sequences were presented in Additional file 1: Table S1.

### MicroRNA prediction

The circRNA/microRNA interaction was predicted with Arraystar’s home-made miRNA target prediction software (Rockville, USA), whose principles are based on the TargetScan and miRanda prediction algorithm. The differentially expressed circRNAs within all the comparisons were annotated in detail with the circRNA/miRNA interaction information.

### MicroRNA target genes prediction and GO analysis

To further investigate the functional roles of microRNA, putative targets of miRNAs were predicted by TargetScan software. GO analysis was performed to explore the functional roles of target genes in terms of biological processes, cellular components and molecular functions. Biological pathways defined by Kyoto Encyclopedia of Genes and Genomes (KEGG), Biocarta and Reactome (http://www.genome.jp/kegg/) were identified by Database for Annotation, Visualization and Integrated Discovery (DAVID; http://www.david.abcc.ncifcrf.gov/).

### CircRNA-microRNA co-expression network

To further elucidate correlations between circRNAs and microRNA, potential microRNA-circRNA-mRNA interaction analysis was conducted by Cytoscape. The size of each node represents the number of putative microRNA functionally connected to each circRNA.

### Statistical analysis

The results were reported as mean ± SD for triplicate measurements. Statistically significant differences between groups were estimated by the Student’s t test using SPSS (13.0). *P* < 0.05 was considered as being statistically significant.

## Result

### Overview of circRNAs profiles

**Fig. 1:**
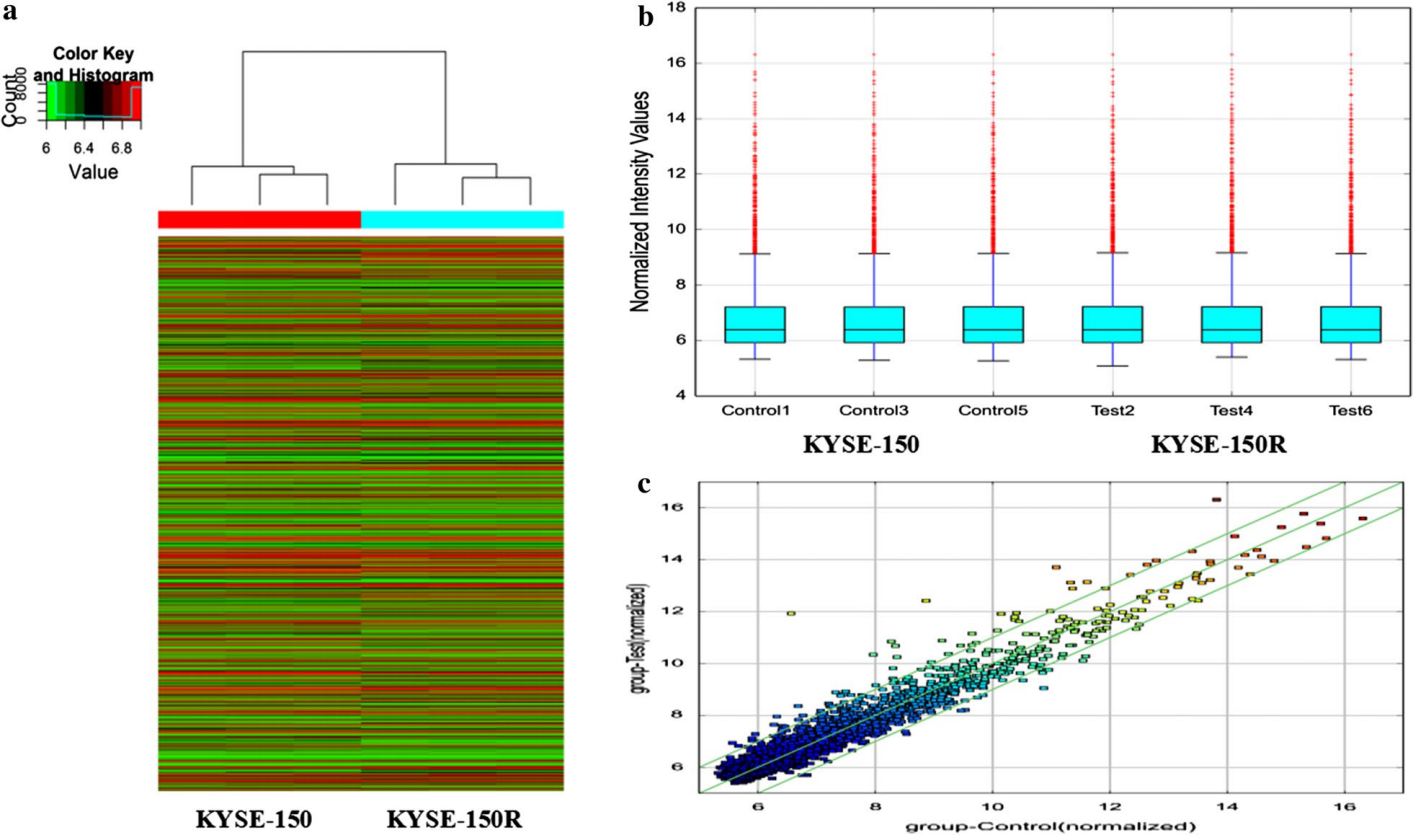
CircRNA microarray expression data between the KYSE-150 and KYSE-150R cells. **a** Hierarchical clustering shows a distinguishable circRNA expression profiling among KYSE-150 and KYSE-150R cell. **b** *Box plots* show the distribution of circRNAs for the two compared samples. The distributions were nearly the same after normalization. **c** *Scatter plots* assess the circRNA expression variation between the two compared groups. The circRNAs above the *top green line* and below the *bottom green line* indicated more than twofold changes of circRNAs between the two compared samples

The expression of 3752 human circRNAs was quantitated for esophageal cancer KYSE-150R and KYSE-150 cell samples using microarray platform (Additional file 2: Table S2). Hierarchical clustering and scatter plot visualization showed that the circRNAs expression levels were distinguishable and variations (Fig. 1). The expression profiles of 74 circRNAs were differentially expressed (fold change ≥2.0 and *P* < 0.05) between the KYSE-150R and KYSE-150 cells, in which 57 circRNAs were found to be upregulated and 17 circRNAs were downregulated more than two-fold in KYSE-150R cells (Tables 1, 2). Among these, The expression levels of circRNA_100385, circRNA_104983 and circRNA_001059 were upregulated in KYSE-150R by 41.06, 11.68 and 6.16-folds, respectively. Meanwhile, circRNA_101877, circRNA_102913, and circRNA_000695 were downregulated by 3.53, 2.69 and 2.51-folds, respectively.

**Table 1:** Upregulated circRNAs in the KYSE-150R compared with KYSE-150

ProbeID	CircRNA	Genesymbol	Chrom	FC (abs)
ASCRP000770	hsa_circRNA_100385	PRRX1	chr1(+):170688866170695542	41.06
ASCRP005230	hsa_circRNA_104983	NHS	chrX(+):1770586117710588	11.69
ASCRP000189	hsa_circRNA_001059	LIN52	chr14(+):7455167774551959	6.16
ASCRP001355	hsa_circRNA_100984	FOXRED1	chr11(+):126142863126143349	5.96
ASCRP000752	hsa_circRNA_100367	DCAF8	chr1(−):160206924160231148	5.62
ASCRP004073	hsa_circRNA_103783	NSUN2	chr5(−):66233266625782	5.19
ASCRP000699	hsa_circRNA_100312	MAN1A2	chr1(+):117944807117984947	5.04
ASCRP005194	hsa_circRNA_104947	PRRC2B	chr9(+):134330462134334743	3.67
ASCRP000700	hsa_circRNA_100313	MAN1A2	chr1(+):117957334117963271	3.46
ASCRP003691	hsa_circRNA_103393	CCDC66	chr3(+):5662699756628056	3.40
ASCRP002905	hsa_circRNA_102592	MYH14	chr19(+):5072087150721028	3.34
ASCRP004116	hsa_circRNA_103826	ZNF131	chr5(+):4316135043162033	3.18
ASCRP004597	hsa_circRNA_104334	MPP6	chr7(+):2466328424708279	3.10
ASCRP004358	hsa_circRNA_104084	LINC00340	chr6(+):2202056722056919	2.96
ASCRP003426	hsa_circRNA_103122	DONSON	chr21(−):3495360734958487	2.91
ASCRP000085	hsa_circRNA_000629	KIF18B	chr17(−):4301230343012398	2.88
ASCRP000698	hsa_circRNA_100311	MAN1A2	chr1(+):117944807117948267	2.88
ASCRP003235	hsa_circRNA_102928	RHBDD1	chr2(+):227729319227779067	2.86
ASCRP001219	hsa_circRNA_100845	MARK2	chr11(+):6366263063663105	2.83
ASCRP002541	hsa_circRNA_102213	USP36	chr17(−):7682332676825089	2.69
ASCRP002519	hsa_circRNA_102191	HN1	chr17(−):7314276073144766	2.67
ASCRP000062	hsa_circRNA_000543	MALAT1	chr11(+):6527249065272586	2.59
ASCRP000292	hsa_circRNA_001543	RSBN1L	chr7(+):7737874077387395	2.58
ASCRP004442	hsa_circRNA_104169	SOBP	chr6(+):107824860107827631	2.56
ASCRP003692	hsa_circRNA_103394	APPL1	chr3(+):5727688357301820	2.54
ASCRP004877	hsa_circRNA_104624	PCMTD1	chr8(−):5277340452773806	2.51
ASCRP000286	hsa_circRNA_001506	LAPTM4A	chr2(−):2024080920240905	2.49
ASCRP001133	hsa_circRNA_100759	DENND5A	chr11(−):92252069229179	2.38
ASCRP001576	hsa_circRNA_101213	RAN	chr12(+):131357380131357465	2.35
ASCRP001668	hsa_circRNA_101309	TMCO3	chr13(+):114188359114193822	2.35
ASCRP003134	hsa_circRNA_102825	RAB3GAP1	chr2(+):135883750135884226	2.33
ASCRP002340	hsa_circRNA_102003	USP22	chr17(−):2091020820914622	2.30
ASCRP004596	hsa_circRNA_104333	MPP6	chr7(+):2466328424690331	2.30
ASCRP000391	hsa_circRNA_100001	SAMD11	chr1(+):871151874792	2.28
ASCRP002927	hsa_circRNA_102614	ASAP2	chr2(+):94909369499018	2.26
ASCRP001679	hsa_circRNA_101320	PRMT5	chr14(−):2339534123396023	2.26
ASCRP002384	hsa_circRNA_102049	TADA2A	chr17(+):3579783835800763	2.25
ASCRP005237	hsa_circRNA_104990	POLA1	chrX(+):2482801424844718	2.25
ASCRP005013	hsa_circRNA_104763	UBAP2	chr9(−):3395328233996331	2.23
ASCRP002333	hsa_circRNA_101996	SPECC1	chr17(+):2010764520109225	2.23
ASCRP003820	hsa_circRNA_103522	FXR1	chr3(+):180685838180688146	2.21
ASCRP001777	hsa_circRNA_101418	CEP128	chr14(−):8129748681307112	2.18
ASCRP002683	hsa_circRNA_102364	DYM	chr18(−):4678337946808545	2.18
ASCRP003659	hsa_circRNA_103361	SMARCC1	chr3(−):4771968747727660	2.18
ASCRP000064	hsa_circRNA_000554	PRB4	chr12(−):1119961811248400	2.17
ASCRP001819	hsa_circRNA_101468	TJP1	chr15(−):3005334130092905	2.16
ASCRP005238	hsa_circRNA_104991	POLA1	chrX(+):2482801424861794	2.15
ASCRP001818	hsa_circRNA_101467	TJP1	chr15(−):3005334130065560	2.13
ASCRP003032	hsa_circRNA_102720	RTN4	chr2(−):5521462655214834	2.12
ASCRP000314	hsa_circRNA_001676	PPP1R12A	chr12(−):8018015380183460	2.11
ASCRP003844	hsa_circRNA_103546	LPP	chr3(+):188202379188242575	2.09
ASCRP001842	hsa_circRNA_101491	MAPKBP1	chr15(+):4210308042105299	2.09
ASCRP002653	hsa_circRNA_102334	SS18	chr18(−):2361236223637706	2.07
ASCRP003119	hsa_circRNA_102810	RALB	chr2(+):121036193121047333	2.06
ASCRP002883	hsa_circRNA_102570	ERCC2	chr19(−):4586052745860957	2.05
ASCRP004893	hsa_circRNA_104640	CSPP1	chr8(+):6801813968028357	2.04
ASCRP003216	hsa_circRNA_102908	BARD1	chr2(−):215632205215646233	2.01

**Table 2:** Downregulated circRNAs in the KYSE-150R compared with KYSE-150

probeID	circRNA	Genesymbol	Chrom	FC (abs)
ASCRP002219	hsa_circRNA_101877	RFWD3	chr16(−):74670243 74671868	3.53
ASCRP003221	hsa_circRNA_102913	ATIC	chr2(+):216177220 216190861	2.69
ASCRP000100	hsa_circRNA_000695	EEFSEC	chr3(+):128102470 128102926	2.51
ASCRP000018	hsa_circRNA_000167	RPPH1	chr14(−):20811404 20811554	2.47
ASCRP001778	hsa_circRNA_101419	STON2	chr14(−):81837331 81837529	2.42
ASCRP001605	hsa_circRNA_101242	PAN3	chr13(+):28830428 28855516	2.39
ASCRP001111	hsa_circRNA_100737	TOLLIP	chr11(−):1307231 1317024	2.3
ASCRP003406	hsa_circRNA_103102	DIDO1	chr20(−):61537238 61545758	2.3
ASCRP001876	hsa_circRNA_101525	PIGB	chr15(+):55621921 55634000	2.25
ASCRP004888	hsa_circRNA_104635	MTFR1	chr8(+):66582107 66582253	2.21
ASCRP003466	hsa_circRNA_103165	PI4KA	chr22(−):21161649 21167794	2.2
ASCRP000390	hsa_circRNA_002178	RPPH1	chr14(−):20811436 20811534	2.16
ASCRP000535	hsa_circRNA_100146	EIF3I	chr1(+):32691771 32692131	2.11
ASCRP000950	hsa_circRNA_100571	PDSS1	chr10(+):27024168 27024508	2.09
ASCRP003989	hsa_circRNA_103695	KLHL8	chr4(−):88116475 88116842	2.07
ASCRP004979	hsa_circRNA_104729	GLIS3	chr9(−):4117767 4118881	2.07
ASCRP001678	hsa_circRNA_101319	RBM23	chr14(−):23378691 23380612	2.01

### Real-time quantitative PCR validation

**Fig. 2:**
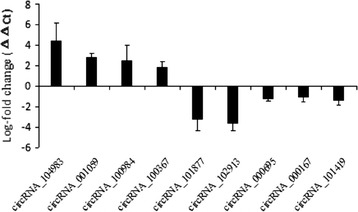
CircRNA expression changes validated by qRT-PCR. Validation of microarray analysis data by qRT-PCR. The expression levels of 9 circRNAs were determined by qRT-PCR. Each qRT-PCR assay was performed at least three times. *P < 0.05

To validate the microarray profiling expression data, real-time quantitative RT-PCR revealed 4 upregulated and 5 downregulated expressed circRNAs as shown in Fig. 2. Expression levels detected by the two methods were consistent with each other, demonstrating the high reliability of the microarray expression results.

### MicroRNA prediction and bioinformatics analyses

**Fig. 3:**
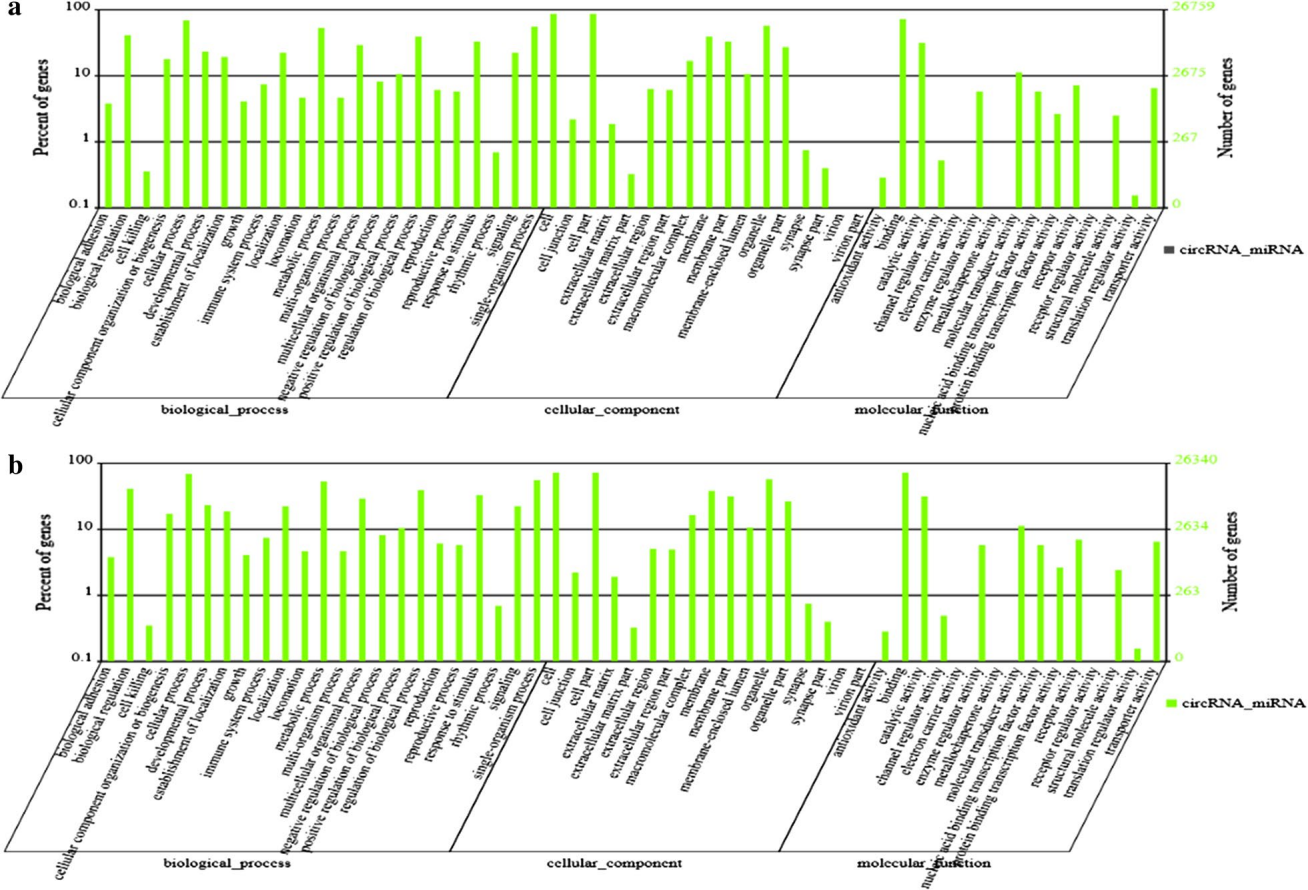
Gene ontology enrichment analysis. **a** Gene ontology enrichment corresponds to the upregulated circRNAs. **b** Gene ontology enrichment corresponds to the downregulated circRNAs

**Fig. 4:**
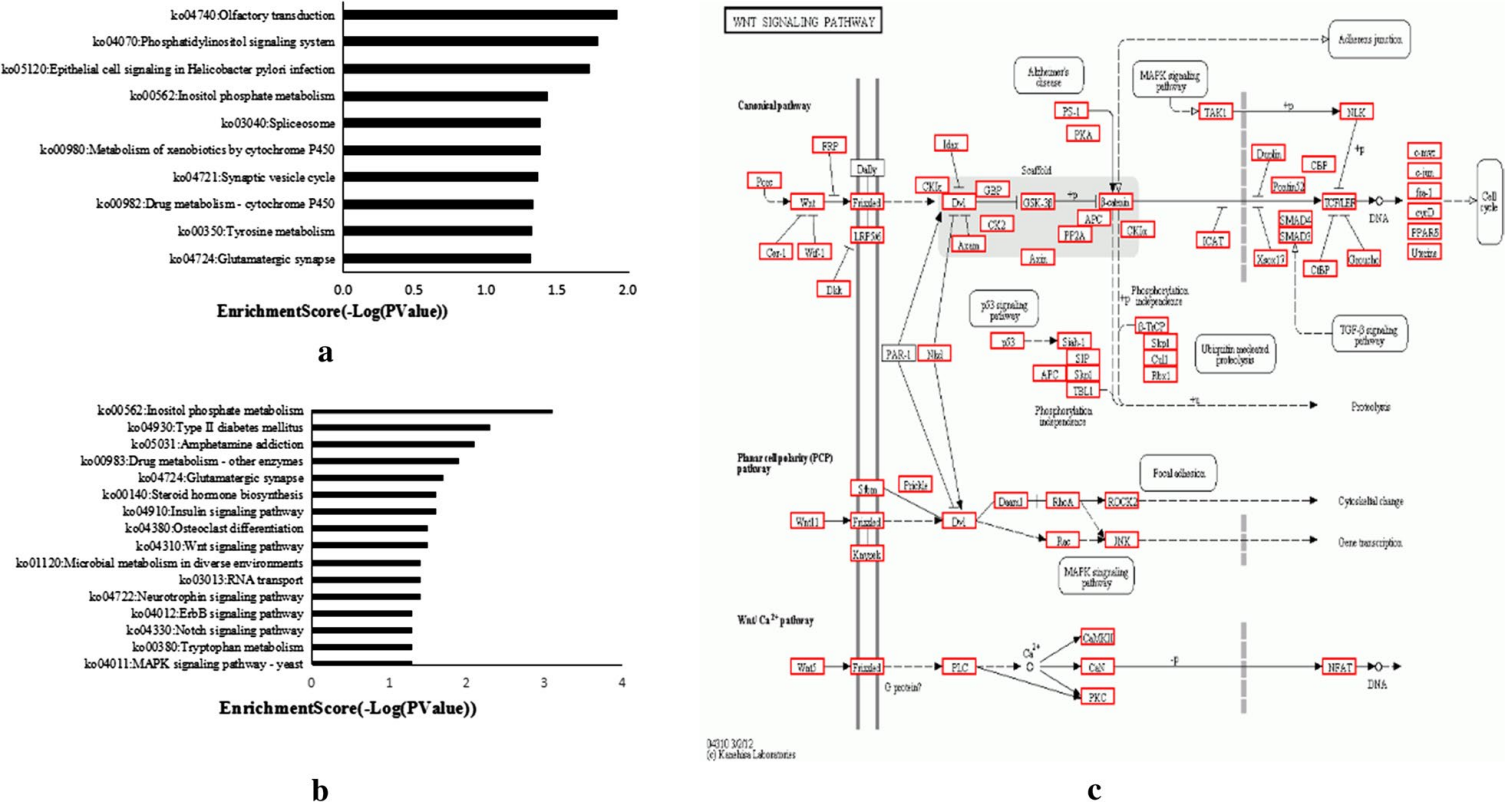
KEGG pathway analysis. **a** Pathways correspond to the upregulated circRNAs. **b** Pathways correspond to the downregulated circRNAs. **c** Wnt signaling pathway. *Red marked* nodes are associated with target genes enrichment on Wnt signaling pathway

MicroRNA prediction was done with Arraystar’s home-made miRNA target prediction software based on miRanda (Table 3; Additional file 3: Table S3, Additional file 4: Table S4). There were 12 out of 17 downregulated circRNAs had target microRNAs. The results of Gene Ontology enrichment analysis on the up and down regulated circRNAs with identified target genes were shown in Fig. 3 and Additional file 5: Table S5. Gene Ontology analysis revealed that numerous target genes were involved in the biological processes, such as cellular process, regulation of biological process, metabolic process, etc. These processes were associated with human tumorigenesis and metastasis. KEGG analysis showed that there were 10 pathways related to upregulated circRNAs, including Olfactory transduction, Spliceosome, Glutamatergic synapse, and Phosphatidylinositol signaling system, and 17 pathways related to downregulated circRNAs, including Neurotrophin signaling pathway, Wnt signaling pathway, Microbial metabolism in diverse environments and Insulin signaling pathway (Fig. 4; Additional file 6: Table S6). Especially, there were more than 400 target genes enrichment in Wnt signaling pathway.

**Table 3:** Target gene numbers of circRNA-miRNA

	CircRNA	MiRNA	MiRNA::circRNA	MiRNA::circRNA target_location	MiRNA::target
Up	57	120	120::53	120::149	120::40335
Down	12	36	36::12	36::12	36::38979

### CircRNA-microRNA co-expression network

**Fig. 5:**
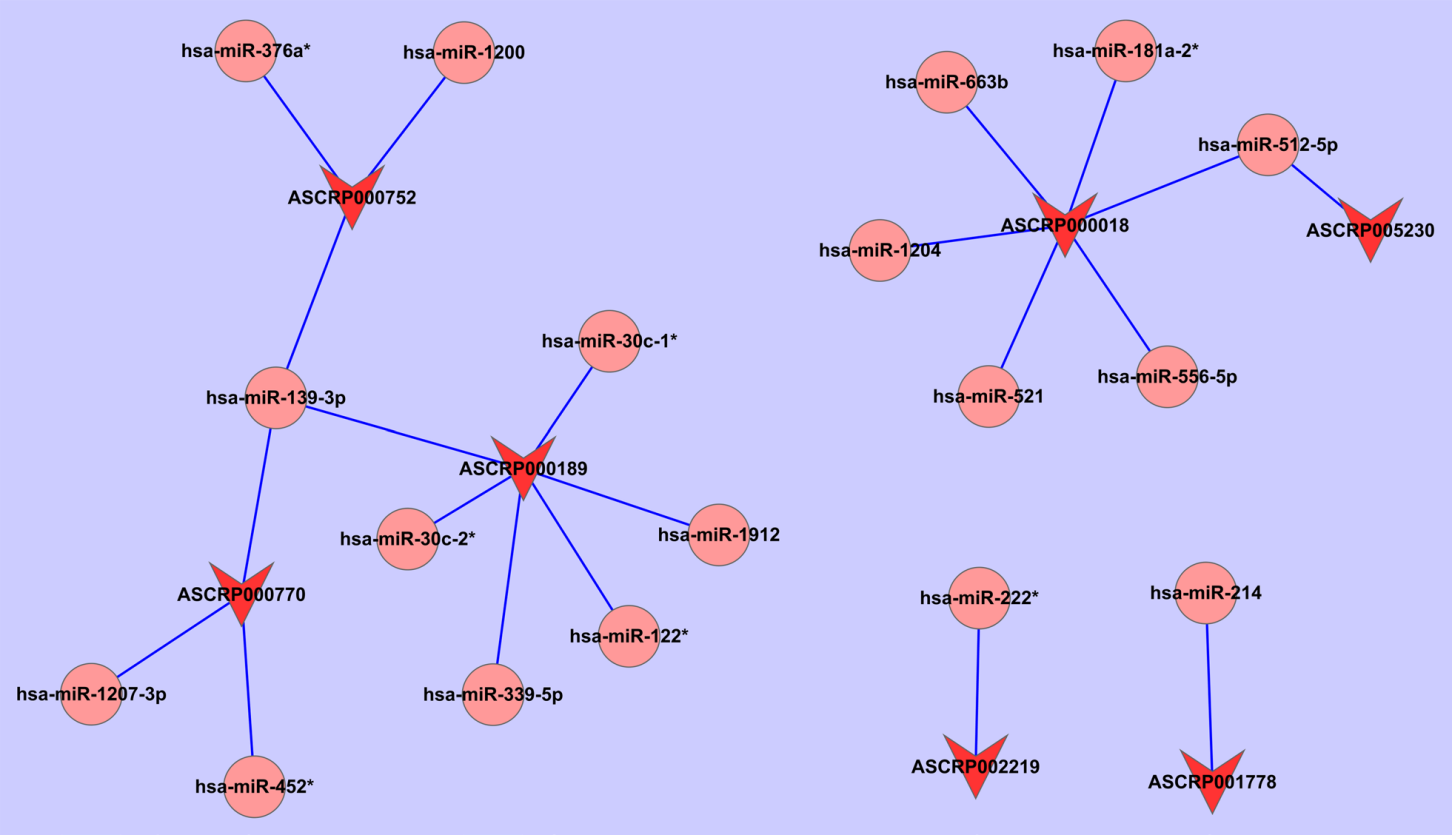
CircRNA-microRNA co-expression network. The circRNA-microRNA co-expression network as drawn with the cytoscape software. The size of each node represent functional connectivity of each circRNA. CircRNA_001059 and circRNA_000167 were the two largest nodes in the network

Potential connections between circRNA and microRNA were explored by using Cytoscape. As shown in Fig. 5, CircRNA_001059 and circRNA_000167 were the two largest nodes in the network.

## Discussion

Acquired radioresistance has been considered as one of the most important reasons causing treatment failure for esophageal cancer patients. In this study, we explored the expression patterns of circRNAs between radioresistant esophageal cancer cell line KYSE-150R and its parental cell line KYSE-150 with Arraystar Circular RNA Microarray to investigate the mechanisms of acquired radioresistance of esophageal cancer. Differentially expressed profiles of circRNAs in radioresistant esophageal cancer cells were observed and validated compared with the parental esophageal cancer cells, indicating possible involvement of these dysregulated circRNAs in the development of radiation resistance of esophageal cancer cells.

CircRNAs are discovered as new special kind of ubiquitous endogenous noncoding RNAs [23]. Recent evidences revealed that circRNAs can function as miRNA sponges and regulate parent gene expression to affect disease. Despite the potential importance of circRNAs reported in several types of cancer [24, 25], there is no reported studies on the functional roles of circRNAs in the radiation resistance of cancer. In this study, there were 57 circRNAs significantly upregulated and 17 circRNAs significantly downregulated in the KYSE-150R cell lines compared with KYSE-150, respectively. In which, circRNA_100385, circRNA_104983 and circRNA_001059 were upregulated with top magnitudes. CircRNA_101877, circRNA_102913, and circRNA_000695 were downregulated with top magnitudes. The expression pattern of these circRNAs were validated by qRT-PCR, and consistent results were observed. Our results indicated that the altered expression levels of circRNAs may be related to their involvement in the transcription level regulation on the radiation resistance of esophageal cancer cells. Aberrant expression of circRNAs has been linked to carcinogenesis and the malignant behavior of many different cancer. The abundance of a circRNA called hsa_circ_002059 has been reported to be significantly downregulated and suggested as a potential diagnostic marker for gastric cancer [26]. Qin M et al. [27] found that hsa_circ_0001649 may play a role in tumorigenesis and metastasis of hepatocellular carcinoma. Li et al. [28] reported that cir-ITCH might influence the expression level of ITCH and may be involved in the development of esophageal squamous cell carcinoma. However, no obvious changes of cir-ITCH was observed in this study, suggesting it may have no contribution to the radioresistance of esophageal cancer cells.

Despite the lack of knowledge of the exact functions of most circRNAs, we investigated the potential targets of these altered miRNAs. In a total, 57 upregulated circRNAs were identified to regulate the expression level of 120 microRNAs using the miRanda software, and 12 downregulated circRNAs were identified to regulate 36 microRNAs. According to the findings of Denzler et al. [29] low levels of circRNAs may not be sufficient to affect the target miRNAs. The circRNA-microRNA co-expression network analysis were conducted for our Top-5 circRNAs in this study. Two potential crucial circRNAs, circRNA_001059 and circRNA_000167 were identified to be influential on target miRNAs. CircRNAs were believed to negatively regulate miRNAs, and contribute substantially to the competing endogenous RNA (ceRNA) network. It has been reported that ciRS-7, as a circular miR-7 inhibitor, harbors more than 60 conventional miR-7 binding sites, which is far more than any known linear sponges [30]. Sex-determining region Y (SRY) was another identified miRNA sponge and functioned as a miR-138 sponge [31]. According to our results, we hypothesized that circRNA_001059 may act as an inhibitor of miRNA by binding several specific miRNAs, including miR-30c-1*, miR-30c-2*, miR-122*, miR-139-3p, miR-339-5p and miR-1912. Our results implied that it is worthwhile to further investigate these novel dysregulated circRNAs as microRNA sponges and their potential biological functions in the development of radiation resistance.

In this study, GO analysis and KEGG pathway annotation were conducted to investigate the functions of related microRNAs [32]. GO enrichment analysis revealed that target genes were involved in the regulation of crucial biological processes, indicating that regulating these genes in the cellular response is of great importance during the development of radioresistance. Among the upregulated pathways found in this study, phosphatidylinositol signaling pathway had been reported to be a key mediator of tumor cell responsiveness to radiation [33]. Phosphatidylinositol 3-kinase (PI3K)/Akt pathway accelerates the repair of DNA-DSB (DNA double-strand breaks), and consequently, its activation leads to therapy resistance [33]. Wnt signaling pathway which corresponds to downregulated circRNAs had also been reported to play predominant roles in radioresistance in Glioblastoma [34] and prostate cancer [35]. These results were also in line with the observations reported in our previous study [21].

## Conclusions

In a conclusion, a unique set of circRNAs and their expression profiles were found in radioresistant esophageal cancer cells. Furthermore, their potential roles were investigated by bioinformatics analysis. Pathway analysis suggested that Wnt signaling pathway may be involved in the radioresistance. Network analysis uncovered two potential key circRNAs, i.e. circRNA_001059 and circRNA_000167. Our results would be helpful for future studies on investigating the molecular functions of circRNAs in radiotherapy resistance.

## Additional files

10.1186/s12967-016-0977-7 circRNAs gene primers in the study for Q-PCR.
10.1186/s12967-016-0977-7 circRNA expression profiling data.
10.1186/s12967-016-0977-7 circRNA_miRNA_pair.
10.1186/s12967-016-0977-7 circRNA_miRNA_target genes.
10.1186/s12967-016-0977-7 circRNA_miRNA.target gene.GO.
10.1186/s12967-016-0977-7 circRNA_miRNA_target gene.pathways.

## References

[1] Conteduca V, Sansonno D, Ingravallo G, Marangi S, Russi S, Lauletta G, Dammacco F (2012). Barrett’s esophagus and esophageal cancer: an overview. Int J Oncol.

[2] Zhang Y (2013). Epidemiology of esophageal cancer. World J Gastroenterol.

[3] Cellini F, Valentini V (2014). Targeted therapies in combination with radiotherapy in oesophageal and gastroesophageal carcinoma. Curr Med Chem.

[4] Sjoquist KM, Burmeister BH, Smithers BM, Zalcberg JR, Simes RJ, Barbour A, Gebski V (2011). Survival after neoadjuvant chemotherapy or chemoradiotherapy for resectable oesophageal carcinoma: an updated meta-analysis. Lancet Oncol.

[5] van Hagen P, Hulshof MC, van Lanschot JJ, Steyerberg EW, van Berge Henegouwen MI, Wijnhoven BP, Richel DJ, Nieuwenhuijzen GA, Hospers GA, Bonenkamp JJ, Cuesta MA, Blaisse RJ (2012). Preoperative chemoradiotherapy for esophageal or junctional cancer. N Engl J Med.

[6] Ding M, Zhang E, He R, Wang X (2013). Newly developed strategies for improving sensitivity to radiation by targeting signal pathways in cancer therapy. Cancer Sci.

[7] Qu S, Yang X, Li X, Wang J, Gao Y, Shang R, Sun W, Dou K, Li H (2015). Circular RNA: a new star of noncoding RNAs. Cancer Lett.

[8] Conn SJ, Pillman KA, Toubia J, Conn VM, Salmanidis M, Phillips CA, Roslan S, Schreiber AW, Gregory PA, Goodall GJ (2015). The RNA binding protein quaking regulates formation of circRNAs. Cell.

[9] Salzman J, Chen RE, Olsen MN, Wang PL, Brown PO (2013). Cell-type specific features of circular RNA expression. PLoS Genet.

[10] Rybak-Wolf A, Stottmeister C, Glažar P, Jens M, Pino N, Giusti S, Hanan M, Behm M, Bartok O, Ashwal-Fluss R, Herzog M, Schreyer L, Papavasileiou P (2015). Circular RNAs in the mammalian brain are highly abundant, conserved, and dynamically expressed. Mol Cell.

[11] Szabo L, Morey R, Palpant NJ, Wang PL, Afari N, Jiang C, Parast MM, Murry CE, Laurent LC, Salzman J (2015). Statistically based splicing detection reveals neural enrichment and tissue-specific induction of circular RNA during human fetal development. Genome Biol.

[12] Chen LL, Yang L (2015). Regulation of circRNA biogenesis. RNA Biol.

[13] Lukiw WJ (2013). Circular RNA (circRNA) in Alzheimer’s disease (AD. Front Genet..

[14] Burd CE, Jeck WR, Liu Y, Sanoff HK, Wang Z, Sharpless NE (2010). Expression of linear and novel circular forms of an INK4/ARF-associated non-coding RNA correlates with atherosclerosis risk. PLoS Genet.

[15] Guo LL, Song CH, Wang P, Dai LP, Zhang JY, Wang KJ (2015). Competing endogenous RNA networks and gastric cancer. World J Gastroenterol.

[16] Li Y, Zheng Q, Bao C, Li S, Guo W, Zhao J, Chen D, Gu J, He X, Huang S (2015). Circular RNA is enriched and stable in exosomes: a promising biomarker for cancer diagnosis. Cell Res.

[17] Hansen TB, Jensen TI, Clausen BH, Bramsen JB, Finsen B, Damgaard CK, Kjems J (2013). Natural RNA circles function as efficient microRNA sponges. Nature.

[18] Ebbesen KK, Kjems J, Hansen TB (2016). Circular RNAs: identification, biogenesis and function. Biochim Biophys Acta.

[19] Hansen TB, Kjems J, Damgaard CK (2013). Circular RNA and miR-7 in cancer. Cancer Res.

[20] Su H, Jin X, Zhang X, Xue S, Deng X, Shen L, Fang Y, Xie C (2014). Identification of microRNAs involved in the radioresistance of esophageal cancer cells. Cell Biol Int.

[21] Su H, Jin X, Zhang X, Zhao L, Lin B, Li L, Fei Z, Shen L, Fang Y (2015). Pan H10, Xie C. FH535 increases the radiosensitivity and reverses epithelial-to-mesenchymal transition of radioresistant esophageal cancer cell line KYSE-150R. J Transl Med.

[22] Su H, Jin X, Shen L, Fang Y, Fei Z, Zhang X, Xie C, Chen X (2015). Inhibition of cyclin D1 enhances sensitivity to radiotherapy and reverses epithelial to mesenchymal transition for esophageal cancer cells. Tumour Biol.

[23] Ivanov A, Memczak S, Wyler E, Torti F, Porath HT, Orejuela MR, Piechotta M, Levanon EY, Landthaler M, Dieterich C, Rajewsky N (2015). Analysis of intron sequences reveals hallmarks of circular RNA biogenesis in animals. Cell Rep.

[24] Bachmayr-Heyda A, Reiner AT, Auer K, Sukhbaatar N, Aust S, Bachleitner-Hofmann T, Mesteri I, Grunt TW, Zeillinger R, Pils D (2015). Correlation of circular RNA abundance with proliferation—exemplified with colorectal and ovarian cancer, idiopathic lung fibrosis, and normal human tissues. Sci Rep.

[25] Qu S, Song W, Yang X, Wang J, Zhang R, Zhang Z, Zhang H, Li H (2015). Microarray expression profile of circular RNAs in human pancreatic ductal adenocarcinoma. Genom Data.

[26] Li P, Chen S, Chen H, Mo X, Li T, Shao Y, Xiao B, Guo J (2015). Using circular RNA as a novel type of biomarker in the screening of gastric cancer. Clin Chim Acta.

[27] Qin M, Liu G, Huo X, Tao X, Sun X, Ge Z, Yang J, Fan J, Liu L, Qin W (2016). Hsa_circ_0001649: a circular RNA and potential novel biomarker for hepatocellular carcinoma. Cancer Biomark.

[28] Li F, Zhang L, Li W, Deng J, Zheng J, An M, Lu J, Zhou Y (2015). Circular RNA ITCH has inhibitory effect on ESCC by suppressing the Wnt/β-catenin pathway. Oncotarget.

[29] Denzler R, Agarwal V, Stefano J, Bartel DP, Stoffel M (2014). Assessing the ceRNA hypothesis with quantitative measurements of miRNA and target abundance. Mol Cell.

[30] Zhao ZJ, Shen J (2015). Circular RNA participates in the carcinogenesis and the malignant behavior of cancer. RNA Biol.

[31] Li J, Yang J, Zhou P, Le Y, Zhou C, Wang S, Xu D, Lin HK, Gong Z (2015). Circular RNAs in cancer: novel insights into origins, properties, functions and implications. Am J Cancer Res.

[32] Gusev Y (2008). Computational methods for analysis of cellular functions and pathways collectively targeted by differentially expressed microRNA. Methods.

[33] Toulany M, Rodemann HP (2015). Phosphatidylinositol 3-kinase/Akt signaling as a key mediator of tumor cell responsiveness to radiation. Semin Cancer Biol.

[34] Kim Y, Kim KH, Lee J, Lee YA, Kim M, Lee SJ, Park K, Yang H, Jin J, Joo KM, Lee J, Nam DH (2012). Wnt activation is implicated in glioblastoma radioresistance. Lab Invest.

[35] Cojoc M, Peitzsch C, Kurth I, Trautmann F, Kunz-Schughart LA, Telegeev GD, Stakhovsky EA, Walker JR, Simin K, Lyle S, Fuessel S, Erdmann K, Wirth MP, Krause M, Baumann M, Dubrovska A (2015). Aldehyde dehydrogenase is regulated by β-catenin/TCF and promotes radioresistance in prostate cancer progenitor cells. Cancer Res.

